# Massively parallel sequencing of 25 autosomal STRs including SE33 in four population groups for forensic applications

**DOI:** 10.1038/s41598-021-82814-z

**Published:** 2021-02-25

**Authors:** Ye-Lim Kwon, Bo Min Kim, Eun Young Lee, Kyoung-Jin Shin

**Affiliations:** 1 Department of Forensic Medicine, Yonsei University College of Medicine, 50-1 Yonsei-ro, Seodaemun-gu, Seoul, 03722 Korea; 2 Brain Korea 21 PLUS Project for Medical Science, Yonsei University, 50-1 Yonsei-ro, Seodaemun-gu, Seoul, 03722 Korea

**Keywords:** Interspersed repetitive sequences, Next-generation sequencing

## Abstract

The introduction of massively parallel sequencing (MPS) in forensic investigation enables sequence-based large-scale multiplexing beyond size-based analysis using capillary electrophoresis (CE). For the practical application of MPS to forensic casework, many population studies have provided sequence data for autosomal short tandem repeats (STRs). However, SE33, a highly polymorphic STR marker, has little sequence-based data because of difficulties in analysis. In this study, 25 autosomal STRs were analyzed, including SE33, using an in-house MPS panel for 350 samples from four populations (African–American, Caucasian, Hispanic, and Korean). The barcoded MPS library was generated using a two-step PCR method and sequenced using a MiSeq System. As a result, 99.88% genotype concordance was obtained between length- and sequence-based analyses. In SE33, the most discordances (eight samples, 0.08%) were observed because of the 4 bp deletion between the CE and MPS primer binding sites. Compared with the length-based CE method, the number of alleles increased from 332 to 725 (2.18-fold) for 25 autosomal STRs in the sequence-based MPS method. Notably, additional 129 unique alleles, a 4.15-fold increase, were detected in SE33 by identifying sequence variations. This population data set provides sequence variations and sequence-based allele frequencies for 25 autosomal STRs.

## Introduction

Short tandem repeats (STRs) are representative markers used for forensic genetic identification and have been traditionally analyzed with capillary electrophoresis (CE)^1^. Recently introduced massively parallel sequencing (MPS), also known as next generation sequencing (NGS), is an alternative technology of CE in that dozens to thousands of forensic markers can be analyzed to the sequence level in a single assay^2,3^. Investigating STRs with MPS is more useful than with CE for challenging casework such as degraded and mixture DNA. Because MPS, unlike CE, has no limit on size of amplicon, it can produce amplicons as small as possible, which is useful for analyzing degraded DNA^4^. Also, MPS has an advantage in paternity testing^5,6^ and mixture deconvolution^7,8^, which require high discrimination power, because of the increased number of alleles observed when considering sequence variation.

As accessibility to MPS has improved in forensic genetics, various MPS–STR multiplex assays have been developed such as the monSTR identity panel^9^, the ForenSeq DNA Signature Prep Kit (Verogen, San Diego, CA, USA) and Precision ID GlobalFiler NGS STR Panel v2 (Thermo Fisher Scientific, Waltham, MA, USA). In 2017, we built an MPS–STR panel that analyzes 25 forensic markers, consisting of 20 expanded CODIS core loci, three additional autosomal STRs (D6S1043, Penta E, and Penta D), and two sex-typing markers (Amelogenin and DYS391), and successfully analyzed the sequence structure of the Korean population^10^. In the present study, this panel has been updated to include three additional loci, SE33, D4S2408, and Y-M175. The upgraded in-house MPS panel simultaneously amplified 28 forensic markers, consisting of 25 autosomal STRs and three sex-typing markers (Amelogenin, DYS391, and Y-M175), but in this study, sequence variation of 25 autosomal STRs will be analyzed. One of the newly added markers, the SE33, is a core locus used with the German national DNA database and is well known to be highly length and sequence polymorphic^11,12^. Since it has been analyzed using various commercial CE panels such as the PowerPlex Fusion 6C (Promega, Madison, WI, USA) and GlobalFiler (Thermo Fisher Scientific), a lot of length-based data have been accumulated. However, because the commercially available MPS panel is limited and SE33 is difficult to analyze, the sequence-based data of SE33 are insufficient compared with other markers^12,13^. In addition, although SE33 is included in the Verogen’s ForenSeq panel, it has been analyzed independently and manually using additional bioinformatics tools such as STRait Razor 2.0 because the ForenSeq Universal Analysis Software (UAS, Verogen) does not support the analysis of SE33^12^. Markers in the in-house MPS–STR multiplex panel were designed to maintain compatibility with the commercial CE kits, the GlobalFiler and the PowerPlex Fusion System (Promega), and to share commonly included markers among three commercially available MPS kits, the ForenSeq DNA Signature Prep Kit, the PowerSeq 46GY System (Promega), and the Precision ID GlobalFiler NGS STR Panel v2.

To apply the MPS method in forensic practice, compilation of sequence data for each population is important^14–17^. In this study, sequence variations of 25 autosomal STRs, including SE33, were analyzed using an in-house MPS panel in four representative populations: African–American, Caucasian, Hispanic, and Korean. Genotype concordance between the CE and MPS methods and the sequence-based structure of STRs were investigated using MPS data analysis pipeline. Further, the significance and utility of the sequence variations detected by MPS will be discussed in the analysis of challenging casework samples.

## Materials and methods

### Samples

The 350 unrelated samples used in this study included four populations: African–American (AfAm, N = 83), Caucasian (Cauc, N = 82), Hispanic (Hisp, N = 82), and Korean (Kor, N = 103). Eighty-three Korean samples were randomly selected from a previous study^10^, and 20 new Korean DNA samples extracted from buccal swabs were added. Informed consent for DNA analysis and research was obtained from all participants over 19 years old. Other population samples were purchased from Coriell Institute Cell Repository (Camden, NJ, USA). All samples were quantified using a Nanodrop 1000 spectrophotometer (Thermo Fisher Scientific) and normalized to 1 ng/μL. This study was approved by the Institution Review Board of Severance Hospital, Yonsei University, Seoul, Korea. All methods were performed in accordance with relevant guidelines and regulations.

### In-house MPS panel for analysis of 25 autosomal STR loci

The in-house multiplex PCR system used in this study was upgraded by adding D4S2408, SE33, and Y-M175 to the customized MPS panel introduced previously^10^. The upgraded system simultaneously amplified 28 markers: 20 expanded CODIS core loci (D1S1656, TPOX, D2S441, D2S1338, D3S1358, FGA, D5S818, CSF1PO, D7S820, D8S1179, D10S1248, TH01, vWA, D12S391, D13S317, D16S539, D18S51, D19S433, D21S11, and D22S1045), five additional autosomal STR loci (D4S2408, D6S1043, Penta E, Penta D, and SE33), and three sex-typing markers (Amelogenin, DYS391, and Y-M175).

PCR primers for D7S820, D8S1179, D13S317, D16S539, and D22S1045 were redesigned to increase PCR yield and eliminate minor PCR interference. Sequence and allele information for the added loci, D4S2408, SE33, and Y-M175, were collected through GenBank (http://www.ncbi.nlm.nih.gov/genbank) and STRBase (http://www.cstl.nist.gov/biotech/strbase), respectively. Primers for targeted markers were designed using Primer3 (http://bioinfo.ut.ee/primer3-0.4.0/primer3/). Primers were designed to avoid variations of > 1% on dbSNP build 153 in binding sites (http://www.ncbi.nlm.nih.gov/projects/SNP/). Information on flanking region SNPs referred to the International Society for Forensic Genetics (ISFG) Guidance^18^ and the Gettings’ study^19^.

### PCR-based MPS library preparation

The MPS library was constructed using two-step PCR, the same strategy used previously^10,20^. First-round PCR generated amplicons using marker-specific primers with read sequences, and the second-round PCR made amplicons using sample-specific indices and platform-specific adaptor sequences. First-round PCR used 20 μL reaction volumes consisting of 1 ng of template DNA, 2 μL of Gold ST*R 10 × Buffer (Promega), 5 U of AmpliTaq Gold DNA Polymerase (Thermo Fisher Scientific), and an appropriate concentration of each primer, as shown in Supplementary Table S1. Thermal cycling was performed on a Veriti 96-Well Thermal Cycler (Thermo Fisher Scientific) at 95 °C for 11 min, 26 cycles at 94 °C for 20 s, 59 °C for 1 min, and 72 °C for 45 s, followed by a final extension at 72 °C for 5 min with a 4 °C soak. Second-round PCR used 20 μL reaction volumes consisting of 1 μL of tenfold diluted PCR product from the first-round, 2 μL of Gold ST*R 10 × Buffer, 5 U of AmpliTaq Gold DNA Polymerase, and 2 μL of Index 1 (i7) and Index 2 (i5) from the Nextera XT index kit v2 (Illumina, San Diego, CA, USA). Thermal cycling was conducted on a Veriti 96-Well Thermal Cycler at 95 °C for 15 min, 15 cycles at 94 °C for 20 s, 61 °C for 30 s, 72 °C for 45 s, and a final extension at 72 °C for 5 min with a 4 °C soak.

### MPS library validation and pooling

Size ranges and concentrations of MPS libraries were confirmed using an Agilent DNA 1000 kit (Agilent Technologies, Santa Clara, CA, USA) on an Agilent 2100 Bioanalyzer (Agilent Technologies). MPS libraries were normalized at 10 ng/μL and purified using 1.2 × Agencourt AMPure XP beads (Beckman Coulter, Indianapolis, IN, USA) according to the manufacturer’s guidelines. The quality of pooled MPS libraries was assessed using an Agilent 2100 Bioanalyzer, and quantity was evaluated using KAPA Library Quantification Kits (Kapa Biosystems, Wilmington, MA, USA) for the Illumina platform on an AB 7500 Real-Time PCR System (Thermo Fisher Scientific) according to the manufacturer’s instruction. Final libraries were normalized to 10 nM.

### MPS data generation and sequence analysis

MPS was performed using a MiSeq Reagent Kit v2 (2 × 250 cycles) or a MiSeq Reagent Kit v3 (2 × 300 cycles) with a MiSeq System (Illumina) according to the manufacturer’s instructions. Pooled libraries were sequenced, and FASTQ files were generated for each sample. FASTQ files were analyzed using STRait Razor 3.0^21^, and the modified configuration file shown in Supplementary Table S2 was used. Minimum coverage (analytical threshold; AT) was set to 5% of each marker. The output file was produced in text format, and it contained the information for sequence-based alleles. All markers, except Y-M175, were analyzed using the read #1 sequence. Output files were analyzed using Microsoft Excel software. The flanking region sequences were acquired from the human reference genome GRCh38/hg38, and STR sequence nomenclature followed ISFG recommendation^18^ (Forensic STR Sequence Structure Guide v5) and Gettings’ report^14^.

### Statistical analysis

Allele frequencies, observed heterozygosity (H_obs_), and expected heterozygosity (H_exp_) were calculated using Microsoft Excel. Likelihood ratio test of linkage disequilibrium (LD) for the syntenic STR loci was performed with Arlequin ver 3.5^22^ (No. of permutations = 10,000, Significance level = 0.05).

### Comparison of STR genotypes between CE and MPS methods

To establish a reference for the MPS data, conventional CE was performed for 350 samples. In this study, each sample was typed using the EzWay Kplex-23 PCR Kit (Komabiotech, Seoul, Korea) and Euplex-13 System, the in-house multiplex PCR kit (the detailed protocol is uploaded at http://forensic.yonsei.ac.kr/protocols.html). Amplicons were separated by size and detected on an AB 3130 Genetic Analyzer (Thermo Fisher Scientific) and genotyped using AB GeneMapper ID Software Version 3.2 (Thermo Fisher Scientific).

Samples with tri-allele were reconfirmed using the PowerPlex Fusion System. MPS was reperformed for the samples with poor sequencing quality. To investigate the cause of the discordance between CE and MPS genotypes, Sanger sequencing was carried out in samples with discordant alleles using sequencing primers with universal primer sequence (M13F/R) in both directions.

## Results and discussion

### Sequencing quality for the in-house MPS panel

The 350 genomic DNA samples from four populations were sequenced using the MiSeq System in several batches. More than 182,000 reads per sample were obtained, and all sequences used for analysis had more than 100 reads. The average depth of coverage (DoC) per marker was minimal for SE33 (3961 reads) and maximal for D2S441 (6703 reads). DoC differences between markers were less than 1.69 × for 25 autosomal STR loci. On average, the allele coverage ratio (ACR) was minimal for Penta E (0.64) and maximal for TH01 (0.86). These show that the in-house MPS panel generates data with even coverage between markers and samples. Detailed information on average DoC and ACR of each marker is presented in Supplementary Figs. S1 and S2.

Moreover, the in-house panel produces amplicons with sizes smaller than 258 bp (all markers were less than 220 bp, except SE33) so that more reliable and accurate sequence data can be obtained from degraded samples^4^, while the amplicon size range for 27 autosomal STRs in the ForenSeq DNA Signature Prep Kit is 61–481 bp^23^. Detailed size range information of our in-house MPS panel is presented in Supplementary Table S3 on the basis of the analyzable range for several MPS assays reported in the Gettings’ study^24^. For each locus, primer binding sites are indicated by hatched lines.

### Concordance of STR genotypes between CE and MPS methods

Concordance rate for 28 forensic markers, 25 autosomal STRs and three sex-typing markers, between CE and MPS methods was 99.88% (9788/9800). The remaining 0.12% (12/9800) genotype discordance was classified into three types. (1) In FGA, one sample (in one allele, 0.01%) showed discordance by allele dropout in MPS result. In this case, heterozygous alleles 20 and 49.2 were genotyped using the CE method. However, using MPS, homozygous allele 20 was genotyped. Dropout allele occurs because of the differences in PCR efficiency between heterozygous alleles with large size differences in the FGA, which has a large allelic range of 14–51.2. In this study, the allele 49.2 was recovered by lowering the threshold (25×), and the sequence structure is as follows; [GGAA]4 GGAG [AAAG]3 [GAAG]4 [AAAG]17 [ACAG]3 [AAAG]12 AA AAAA [GAAA]4. (2) In vWA, three samples (in three alleles, 0.03%) displayed discrepancies by dropout of MPS-based allele. As a result of Sanger sequencing, the flanking region SNP rs771794429 was detected at the MPS primer binding site of the dropped out alleles in vWA. The SNP was found in alleles 14 and 15 in African–American samples on our dataset and also mainly observed in alleles 12–15 in African–American samples on other studies^14,15^. Furthermore, Devesse et al*.*^25^ reported the SNP in alleles 13–15 in only West African samples, not North East African. This implicates that the rs771794429 is potentially African population specific and might be linked with specific alleles. In this respect, caution is required when analyzing the vWA for African samples using the MPS panel suggested in this study. (3) In SE33, eight samples (in nine alleles, 0.08%) represented genotype discordance. In one sample, alleles 12 and 17 were genotyped by CE. However, alleles 13 and 17 were genotyped by MPS. A 4 bp deletion (rs369314007) was observed between CE and MPS primer binding sites by Sanger sequencing. All other samples also presented 4 bp deletions between CE and MPS primer binding sites in the 3′ flanking region. Some polymorphisms might be observed between CE and MPS primer binding sites, because the CE amplicon size was designed to be large in the GlobalFiler (307–438 bp)^26^ and Euplex-13 (171–321 bp) systems, while the in-house MPS amplicon size was designed to be as small as possible (120–258 bp). The 4 bp deletion caused the sequence-based alleles to be one repeat larger than length-based alleles. Of nine alleles, four alleles showed [TTTT/–] deletion (rs369314007), and five alleles showed [TCTT/–] deletion (rs1371483225). In one case, these two 4 bp deletions (rs369314007 and rs1371483225) occurred simultaneously in each heterozygous allele of one sample (CE-based alleles are 16 and 23.2, and MPS-based alleles are 17 and 24.2). The two 4 bp deletions (rs369314007 and rs1371483225) that produced the genotype discordance observed in this study were reported by Borsuk et al*.*^12^. The discordant genotypes of SE33 are fully explained by deletions in the flanking region. Considering these 4 bp deletions, the sequence- and length-based alleles show 100% concordance in SE33. Detailed allele discrepancy information is provided in Supplementary Table S4.

Fifteen tri-allelic genotypes were observed in 350 samples for 25 autosomal STR loci: four at SE33, three at D18S51, two at FGA, and one at D2S1338, D4S2408, D8S1179, D12S391, Penta E, and D16S539. Each tri-allele was identified by more than one method, and detailed information is provided in Supplementary Table S5.

### Sequence variation and allele gain

All sequence structures observed in this study are presented in Supplementary Table S6 and were analyzed by referring to the Supplementary Table S3 of the Gettings’ report^14^. Supplementary Table S6 contains not only the sequence structures but also the calculated sequence-based allele frequencies in four populations (African–American, Caucasian, Hispanic, and Korean). Conversion of sequences to length-based alleles for STR allows for backward compatibility with conventional CE-based data. Excluding the four dropped out alleles in FGA and vWA, the total number of alleles for the 25 autosomal STRs across four populations increased 2.18-fold. (The total number of length-based alleles was 332, and that of sequence-based alleles was 725.)

**Figure 1:**
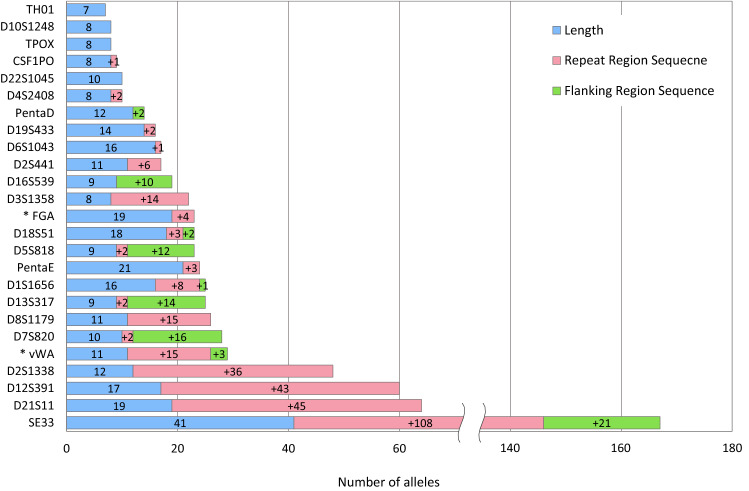
Number of length- and sequence-based alleles for 25 autosomal short tandem repeats (STRs) across four populations (N = 350). Dropped out alleles in FGA and vWA were not included, and they are marked with *. The length-based alleles are in blue boxes, the sequence-based alleles by repeat region variation are in pink boxes, and the sequence-based alleles by flanking region variation are in green.

Figure 1 shows the number of length- and sequence-based alleles for each marker in four populations and the allele gain by sequence in repeat and flanking regions. These results are similar to previous studies using several commercial MPS panels^14–17,27,28^. Four STR loci (TPOX, TH01, D10S1248 and D22S1045) of the 25 autosomal STRs showed no gain in the number of alleles by sequence. Of the remaining twenty-one STR loci, nineteen were distinguished by repeat region variation, and nine were identified by flanking region variation. Repeat region variations are mainly observed in SE33, D21S11, D12S391, and D2S1338. Most flanking region variations are represented at SE33, D7S820, D13S317, D5S818, and D16S539. Notably, SE33 has significant repeat and flanking region variations. By identifying sequence variations of SE33, additional 129 unique alleles were detected, a 4.15-fold increase. D21S11, D12S391, and D2S1338 showed more than three-fold increases in the number of observed alleles. Four markers increased more than three-fold (SE33, D21S11, D12S391, and D2S1338) are complex/compound repeats. Owing to the combination of the number of repeat units and variations in the repeat region, complex/compound repeats showed a large increase in the number of unique sequence-based alleles. By contrast, simple repeats such as TH01, D10S1248, TPOX, and D22S1048 had few or no increased numbers of alleles by sequence.

Detailed information on the number of observed alleles for each population is presented in Supplementary Figs. S3–S6. SE33 showed the greatest increase (2.5-fold or more) in observed allele numbers for each population. In addition, the rate of increase in the number of alleles by repeat region variations in SE33 and D2S1338 markers was higher in the Korean and African–American populations, respectively, compared with that in other populations.

Thirty-one flanking region variations were observed at nine autosomal STR loci from all populations: seven at SE33, six at D13S317, five at D7S820, four at D16S539, three at Penta D, two each at D5S818 and vWA, and one each at D1S1656 and D18S51 (see Supplementary Table S7). Specifically, variations at the SE33 locus consisted of four SNPs and three deletions, along with the highest number of flanking region variations observed. In D1S1656, all the alleles with rs4847015 are 0.3 microvariant and are composed of the same repeat motif: CCTA [TCTA]n TCA [TCTA]n (alleles 14.3–19.3). This shows a potential linkage between a specific SNP and repeat sequence variant^29^.

The sequence structure obtained by the MPS method was generalized to the motif based on the variation observed in the repeat and flanking regions (see Supplementary Table S8), as reported by Gettings et al*.*^14^. In general, variable stretches are indicated with “n”, and motifs with frequencies below 1% in all populations are classified as “all other motifs”. The differences in motif frequency of over 20% from other populations are indicated in red letters. Motif frequencies of [TCTA]n TCTG TCTA for D2S441 in Hispanic and TCTA TCTG [TCTA]n for D3S1358 in African–American populations were relatively high compared to others. By contrast, motif frequencies of [TCTA]n for D8S1179 in African–American and [AGAT]n [AGAC]n AGAT for D12S391 in Caucasian populations were relatively low.

Of the three additional sex chromosome markers, DYS391 and Y-M175 markers showed no sequence variations, and the amelogenin Y had a SNP (rs375383821) in the African–American population. This SNP was confirmed to be African-specific by gnomAD.

### Length- and sequence-level analysis for SE33

**Figure 2:**
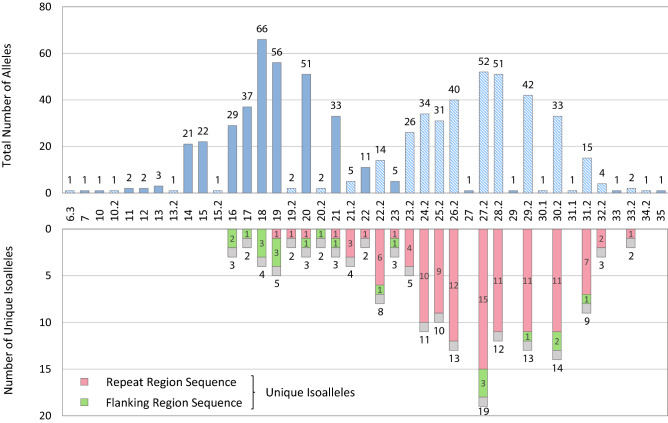
Allele distribution of SE33. The upper graph displays the total numbers of alleles by length. Microvariant alleles are indicated by hatched lines. The lower graph represents the number of isoalleles by variations in repeat and flanking regions.

The allele distribution pattern of SE33 is shown in Fig. 2. In the upper graph, a pattern of the total number of alleles by length appears bimodal distribution, which is similar to previous studies^12^. In this graph, integer alleles were mainly observed in small allele (particularly allele 18) and microvariant alleles were shown in large allele (particularly allele 27.2). The lower graph shows the number of unique isoalleles – alleles of the same length but different sequence. For example, allele 22 has two unique isoalleles (CTTC [CTTT]21 rs9362477 and [CTTT]22 rs9362477), which are distinguished by repeat region variation. According to the Supplementary Table S8, the repeat structure of SE33 is basically [CTTT]n. Different repeat structures are observed as this motif is interrupted by TT/CT, and/or the first repeat motif is changed to CTTC; [CTTT]n TT/CT [CTTT]m and/or CTTC [CTTT]n. The small sized alleles are mainly integer and have [CTTT]n motif, and the large sized alleles are usually 0.2 microvariant and have [CTTT]n TT/CT [CTTT]m motif. The large alleles have more isoalleles because they have unique structures depending on the combination (n, m) of the number of repeat units before and after the TT/CT.

**Figure 3:**
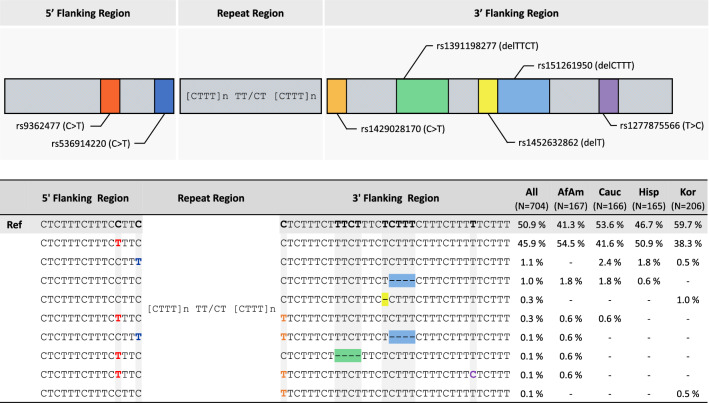
Flanking region polymorphisms in SE33. The upper figure shows the relative positions of the seven polymorphisms observed in the flanking region of SE33. The lower table represents the sequence-based allele frequencies separated by flanking region polymorphisms for each population (N, the number of alleles observed in SE33): Red, rs9362477 (C>T); Navy, rs536914220 (C>T); Orange, rs1429028170 (C>T); Green, rs1391198277 (delTTCT); Yellow, rs1452632862 (delT); Blue, rs151261950 (delCTTT); Purple, rs1277875566 (T>C).

Figure 3 shows the flanking region polymorphisms observed when analyzing sequence-based SE33 in four populations using the in-house MPS panel (Details on the position of the polymorphisms are presented in Supplementary Table S7). Additional nine unique sequences were obtained by analyzing the flanking region sequence, and further identification can be performed for half out of observed alleles across four populations. Considering these flanking region polymorphisms and the repeat region variation together, SE33 would be a more polymorphic marker and useful for human identification and paternity testing.

### Population statistics

In Supplementary Table S9, expected heterozygosity and observed heterozygosity were calculated based on the allele frequencies obtained by length- and sequence-based methods. By analyzing STRs at sequence-level, seven STRs are more than 90% in H_exp_, while only two STRs with more than 90% H_exp_ are observed in length-based analysis. SE33 showed the highest heterozygosity (97.3%) in sequence-based analysis, followed by D21S11 (93.0%), D2S1338 (92.4%), and Penta E (92.3%). By contrast, the highest heterozygosity increase between the CE and MPS methods was in D3S1358 (75.8% > 91.6%), followed by D5S818, D21S11, and D8S1179.

Supplementary Table S10 contains LD *p* values for the seven syntenic pairs in each population: TPOX-D2S441, D2S441-D2S1338, D4S2408-FGA, D5S818-CSF1PO, D6S1043-SE33, vWA-D12S391, and D21S11-PentaD. Among these seven syntenic pairs, significant level of LD (*p* < 0.05) were observed at TPOX-D2S441 in the Korean population (*p* value = 0.0279) and vWA-D12S391 in the Hispanic population (*p* value = 0.0388), but no significant LD was detected after Bonferroni correction. However, because D6S1043 and SE33 are very close in that the physical distance of two markers is 3.46 Mb and the recombination fraction is 0.0440^30^, it is recommended that only one of the profiles obtained from both markers is used when analyzing kinship.

## Conclusion

Twenty-five autosomal STRs were successfully analyzed, including SE33, for 350 samples across four representative populations using the upgraded in-house MPS panel. Compared to standard CE method, the total number of alleles increased 2.18-fold in the MPS method. Identifying sequence variations in repeat and flanking regions of STRs by using MPS enhances the power of discrimination. Especially, sequence-based analysis of SE33 additionally had 129 unique alleles, which showed a 4.15-fold increase. SE33 is highly polymorphic and useful not only for forensic human identification but also for complex kinship tests. The in-house MPS panel, which generated a well-balanced depth of coverage and short sized amplicons, is promising in the analysis of degraded DNA. In addition, if a more user-friendly MPS data analysis pipeline is available, it will encourage a researcher or practitioner to easily accumulate sequence-based STR data for worldwide populations and facilitate the adoption of MPS in the forensic genetics.

## Supplementary Information

Supplementary Figures.

Supplementary Table 1.

Supplementary Table 2.

Supplementary Table 3.

Supplementary Table 4.

Supplementary Table 5.

Supplementary Table 6.

Supplementary Table 7.

Supplementary Table 8.

Supplementary Table 9.

Supplementary Table 10.

## Data Availability

The datasets analyzed during the current study are available upon request by contact with the corresponding author (Kyoung-Jin Shin, KJSHIN@yuhs.ac).
