# Supplementary material for: Massively parallel sequencing of 25 autosomal STRs including SE33 in four population groups for forensic applications
Source: Sci Rep. 2021 Feb 25;11:4701. doi: 10.1038/s41598-021-82814-z (PMC7907369; doi:10.1038/s41598-021-82814-z)
Supplement: Supplementary file 1 — Supplementary Figures. [file 41598_2021_82814_MOESM1_ESM.pdf]

Supplementary Figures

Massively parallel sequencing of 25 autosomal STRs including SE33 in four population groups for forensic applications

Ye-Lim Kwon[1,2], Bo min Kim[1,2], Eun Young Lee[1], and Kyoung-Jin Shin[1,2,*]

[1]Department of Forensic Medicine, Yonsei University College of Medicine, 50-1 Yonsei-ro, Seodaemun-gu, Seoul 03722, Korea

[2]Brain Korea 21 PLUS Project for Medical Science, Yonsei University, 50-1 Yonsei-ro, Seodaemun-gu, Seoul 03722, Korea

*Corresponding author at: Department of Forensic Medicine, Yonsei University College of Medicine, 50-1 Yonsei-ro, Seodaemun-gu, Seoul 03722, Korea

E-mail address: KJSHIN@yuhs.ac (K-J. Shin)

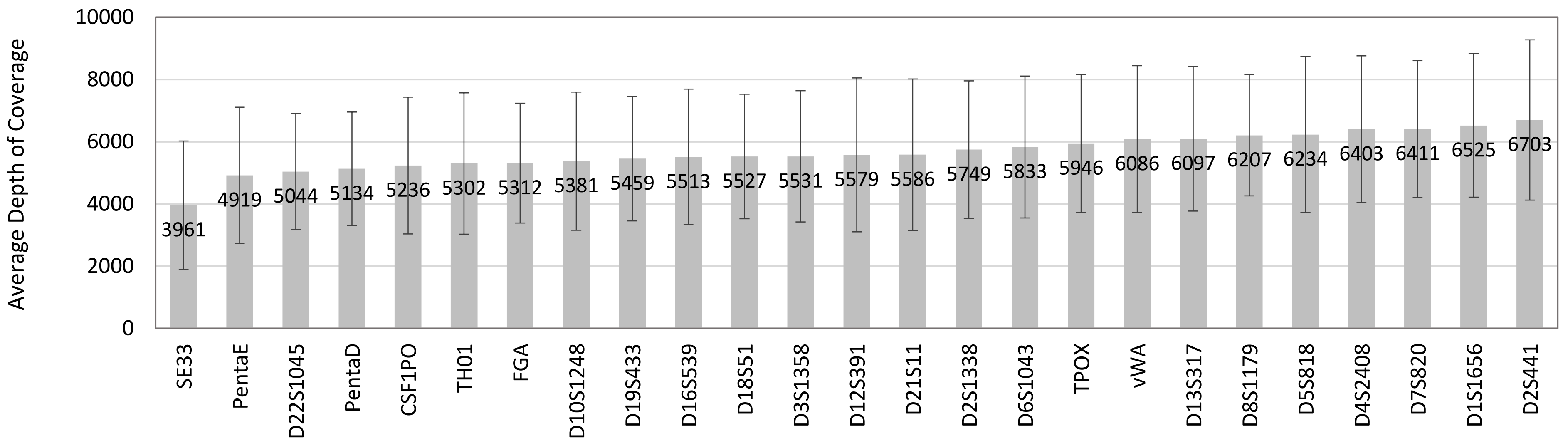

Supplementary Fig. S1 Average depth of coverage (DoC) for 25 autosomal short tandem repeats (STRs) across four populations.

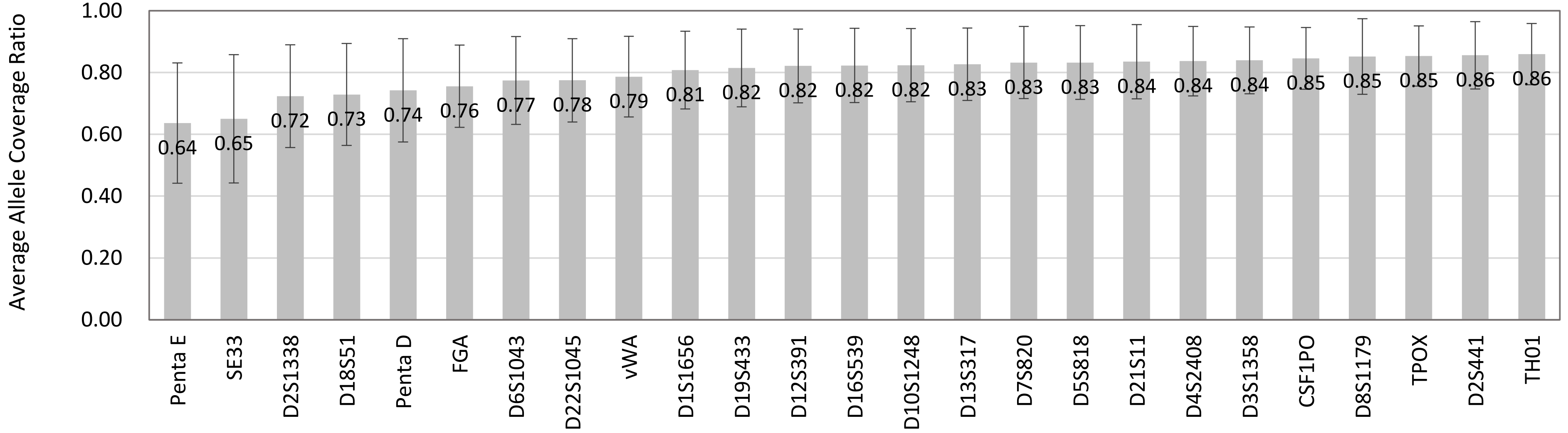

Supplementary Fig. S2 Average allele coverage ratio (ACR) for 25 autosomal short tandem repeats (STRs) across four populations.

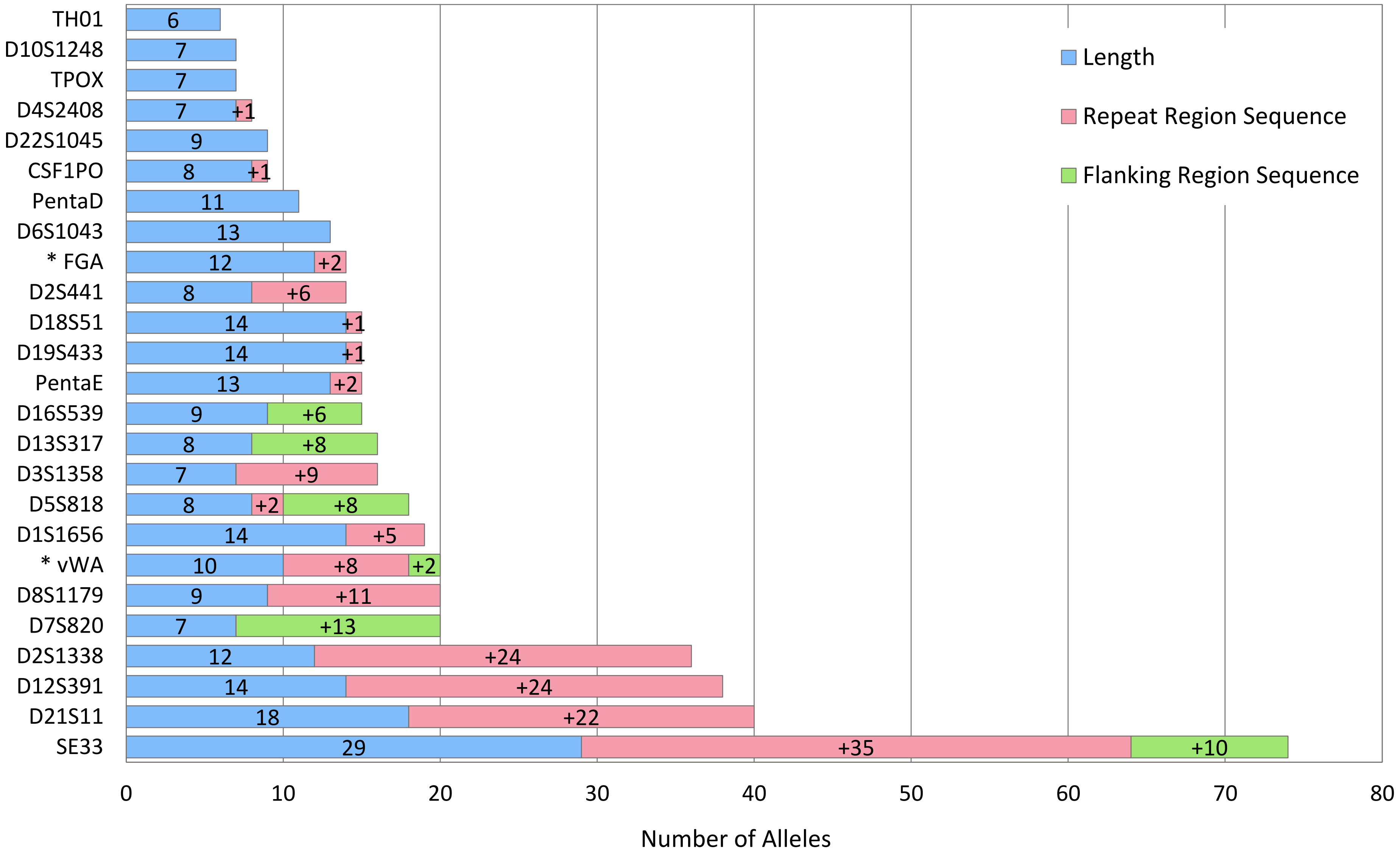

Supplementary Fig. S3 Number of length- and sequence-based alleles for 25 autosomal short tandem repeats (STRs) in African–American (N = 83). Dropped out alleles in FGA and vWA were not included, and they are marked with *. The length-based alleles are in blue boxes, the sequence-based alleles by repeat region variation are in pink boxes, and the sequence-based alleles by flanking region variation are in green.

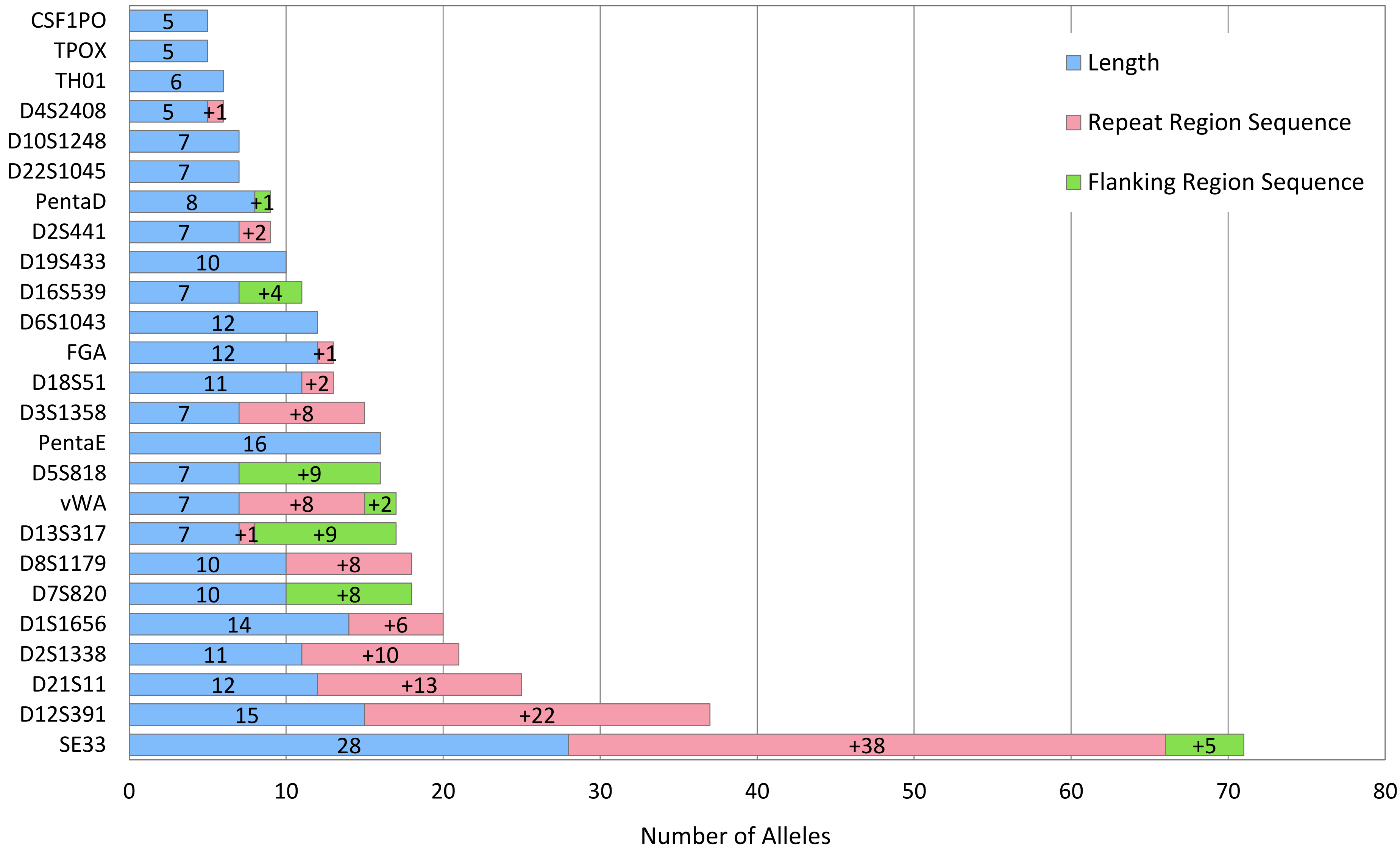

Supplementary Fig. S4 Number of length- and sequence-based alleles for 25 autosomal short tandem repeats (STRs) in Caucasian (N = 82). The length-based alleles are in blue boxes, the sequence-based alleles by repeat region variation are in pink boxes, and the sequence-based alleles by flanking region variation are in green.

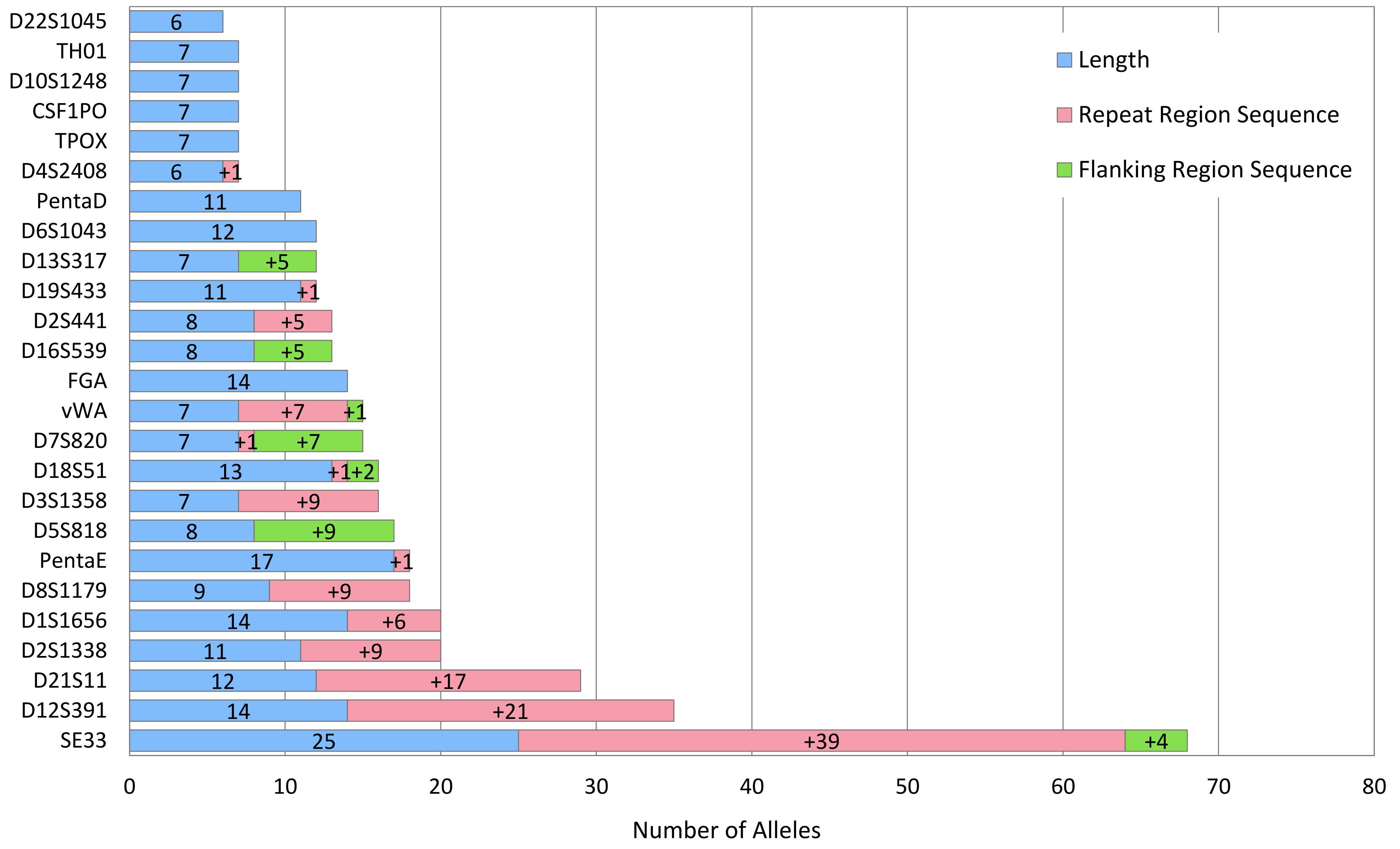

Supplementary Fig. S5 Number of length- and sequence-based alleles for 25 autosomal short tandem repeats (STRs) in Hispanic (N = 82). The length-based alleles are in blue boxes, the sequence-based alleles by repeat region variation are in pink boxes, and the sequence-based alleles by flanking region variation are in green.

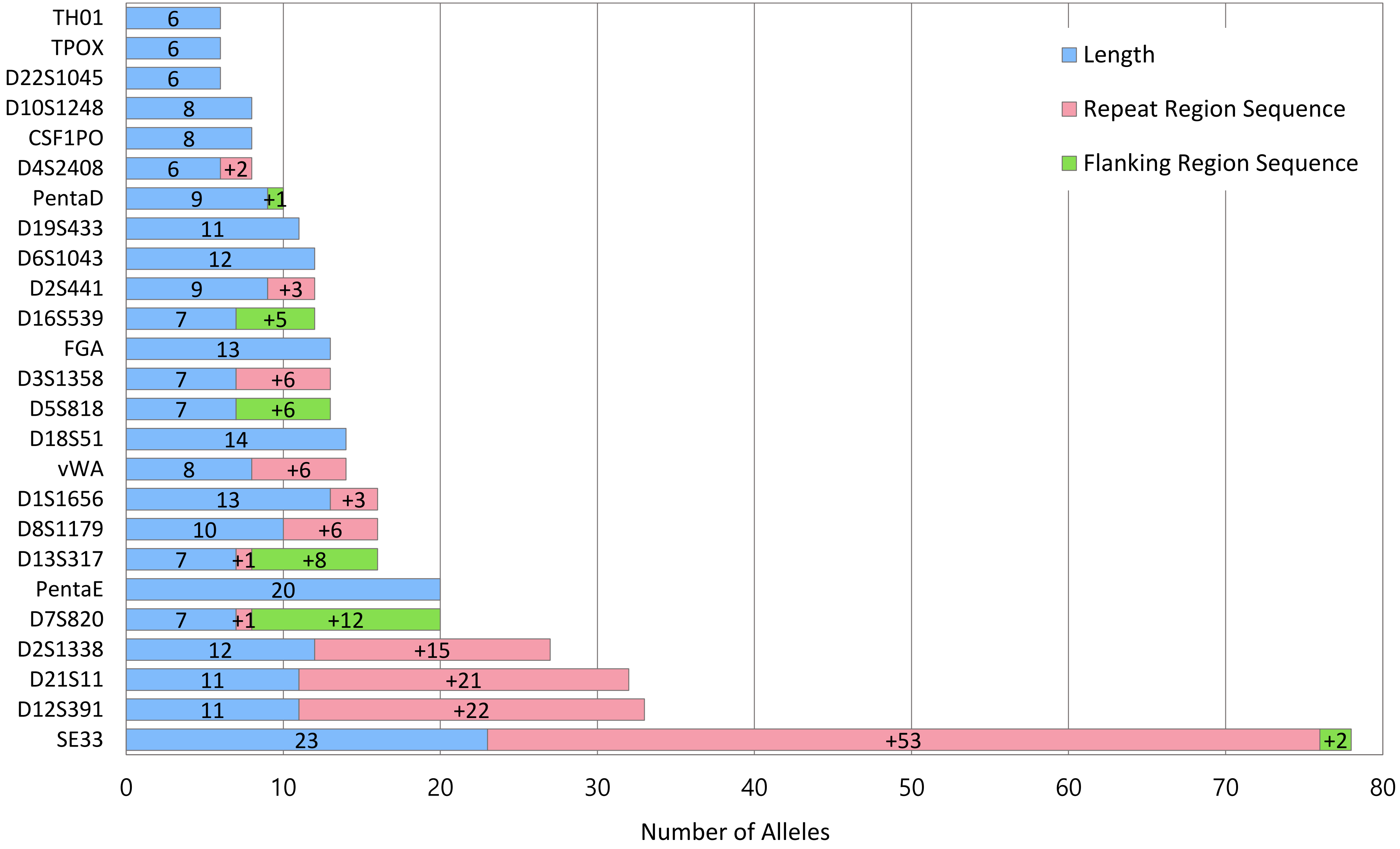

Supplementary Fig. S6 Number of length- and sequence-based alleles for 25 autosomal short tandem repeats (STRs) in Korean((N = 103). The length-based alleles are in blue boxes, the sequence-based alleles by repeat region variation are in pink boxes, and the sequence-based alleles by flanking region variation are in green.
